# 

**Published:** 1840

**Authors:** 


### BIBLIOGRAPHICAL RECORD.

1. First Annual Report of the Crighton Royal Institution for Lunatics, Dumfries. June, 1840.

---

2. Cyclopædia of Anatomy and Physiology. Edited by Dr. Todd. Part 19. July, 1840.

---

3. The Cyclopædia of Practical Surgery, &c. Edited by W. Costello, M.D. &c. Part VI. June, 1840.

---

4. Trifles from my Portfolio, or Recollections of Scenes and Small Adventures during 29 years' Military Service, &c. &c. By a Staff-Surgeon. Two Vols. 8vo. Quebec, 1839. Highley, London, 1840.

☞ *Very amusing and interesting reminiscences.*

---

5. Library of Medicine. By Alex. Tweedie, M.D. Vol. IV. and V. Whittaker and Co. 1840.

---

6. The Anatomists Vade Mecum: a System of Human Anatomy. By W. J. Erasmus Wilson. With upwards of 150 illustrations by Bagg. Octavo, pp. 551. Churchill, London, 1840.

---

7. The Ceylon Moss. Communications read to the Royal Medico-Botanical Society of London. By George G. Sigmond, M.D. and Fred. Farre, M.D. 8vo. pp. 85. Renshaw, London, 1840.

---

8. The Horley-Green Mineral Water: its Chemical Analysis and Medicinal Uses. By W. Alexander, M.D. Physician to the Halifax Infirmary, &c. 8vo. pp. 50. Longman and Co. 1840.

9. Return: Statistical Reports on the Health of the Navy, for the years 1830-1837, inclusive. South American, West Indian, and North American, Mediterranean, and Peninsular Commands. Ordered by the House of Commons, 1840. (Price 3s. 9d.)

---

10. Lectures on the Morbid Anatomy of the Serous and Mucous Membranes. In 2 Vols. By Thomas Hodgkin, M.D. &c. &c. Vol. II. Part I. Mucous Membranes. pp. 551. Simpkin and Co. London, 1840.

☞ *We are glad to see our talented friend returned to active practice again, and hope he may long continue in it.*

---

11. The Retrospective Address, delivered at the Seventh Anniversary Meeting of the Provincial Medical and Surgical Association, held at Liverpool July 24th and 25th, 1839. By John Addington Symonds, M.D. Senior Physician to the Bristol Hospital, &c. &c. 8vo. pp. 100. Worcester, 1840.

---

12. On the Treatment of certain Injuries of the Eye, occurring in Infants and Young Persons. By Richard Middlemore, Esq. Surgeon to the Birmingham Eye Infirmary.

---

13. A Letter to James Thomas Law, M.A. Chancellor of the Diocese of Litchfield, on the Importance of Establishing, in connexion with the Birmingham Royal School of Medicine and Surgery, a Clinical Hospital. By William Sands Cox, F.R.S. &c. 8vo. pp. 28. Birmingham, 1839.

---

14. A Supplement to his Statements to Mr. Warburton in the Parliamentary Committee of the House of Commons in 1834, also to his Replies to the Poor-Law Commissioners of Inquiry, in 1836. With an Appendix. By William Stoker, M.D. &c. 8vo. pp. 22, appendix pp. 12. Dublin, 1840.

---

15. The Aortic System, Anatomically and Physiologically considered, with a view to set forth, by Instance or Example, the Wisdom, Power, and Goodness of God, as revealed and declared in Holy Writ. The Warneford Prize Essay, for the year 1839. By Edward Smith, Student of the Birmingham Royal School of Medicine and Surgery. 8vo. pp. 91. London and Birmingham, 1840.

---

16. An Essay on the Treatment and Cure of Pulmonary Consumption, on principles natural, rational, and successful: with Suggestions for an improved Plan of Treatment of the Disease amongst the Lower Classes of Society; and a Relation of several successive Cases restored from the last Stage of Consumption to a good state of health. By George Bodington, Surgeon. 12mo. pp. 60. London: Longman & Co. 1840.

---

17. A Letter on the Efficacy of Mineral Waters in the Treatment of Chronic Disorders. By James Pickford, Esq. Brighton 1840. Price 1s.

---

18. The Phrenological Journal for April 1, 1840, and also for July 1, 1840.

---

19. Dr. Brainard's Address to the Candidates for Degrees and Licences in the Medical Institution of Vale College, Jan. 1, 1840.

---

20. A Report upon Deafness, when resulting from Diseases of the Eustachian Tube, with the Modern Method of Cure, &c. being a Paper read at the Liverpool Medical Association, 1839. By Hugh Neill, Surgeon. Liverpool, 1840.

---

21. The Maternal Management of Children in Health and in Disease. By Thos. Bull, M.D. &c. Longman and Co. 1840.

---

22. Acute Hydrocephalus, or Water in the Head, an Inflammatory Disease, and Curable equally and by the same means as other Diseases of Inflammation. By David D. Davis, M.D. Professor of Obstetric Medicine in the University College, &c. 8vo. pp. 309. Taylor and Walton, London, 1840.

---

23. An Address upon Laying the Foundation Stone of the Queen's Hospital, Birmingham, June 18, 1840. By Vaughan Thomas, B.D. Oxford, 1840.

---

24. The Cyclopædiæ of Anatomy and Physiology. Edited by Robert B. Todd, M.D. F.R.S. Part XX. London, Sherwood and Co. 1840.

---

25. An Essay on Intoxicating Liquors, &c. By Mr. T. Herbert Baker, Surgeon, Bedford. London, Hamilton, Adams and Co. 1838.

---

26. Medical and Physiological Commentaries. By Martyn Paine, M.D. A.M. In two vols. New York, Collins, Keese, and Co. 1840. London, Churchill, Princes-street, Soho.

---

27. On the best Method of Obtaining the more powerful Vegetable Preparations for Medical Use. By Edward Bentley, Operative Chemist.

---

28. A History of British Birds. By William Yarrell, F.L.S. Parts 18 and 19. J. Van Voorst, 1, Paternoster-row, June and July, 1840.

---

29. A General Outline of the Animal Kingdom, and Mauual of Comparative Anatomy. By Thomas Rymer Jones, F.Z.S. Professor of Comparative Anatomy in King's College, London. Parts X. and XI. J. Van Voorst, Paternoster-row, 1840.

---

30. Surgical, Operative, and Mechanical Dentistry:—the Substance of a Series of Lectures delivered by L. Charles de Loude, &c. 8vo. pp. 196, with a Plate. Whittaker, London, 1840.

---

31. The Retrospect of Practical Medicine and Surgery, for the first six months of the year 1840, &c. By W. Braithewaite, Esq. Vol. 1, January to July, 1840. Simpkin and Marshall, 1840.

---

32. A Treatise on the Eye, containing Discoveries of the Causes of Near and Far Sightedness; and of the Affections of the Retina, with Remarks on the Use of Medicines as substitutes for Spectacles. By W. Chas. Wallace, Oculist. Second Edition, New York, 1839.

